# The Waste Places

**Published:** 1917-02

**Authors:** 


### The Waste Places.

Mary had a little waist,
'Twas puzzling to her beau,
For everywhere the fashion went,
Her waist was sure to go.
Sometimes it was beneath her arms,
Sometimes below her knee,
Sometimes she had no waist at all
So far as he could see.

—*Burt's Box Bulletin.*

Any person that is hard of hearing can secure literature that may prove helpful, by addressing the Volta Bureau, 1601 Thirty-fifth Street, N. W., Washington, D. C. We do not give medical advice, we have no medicines or instruments for sale, and we do no teaching.

